# The transition from tube feeding to oral feeding algorithm in preterm infants: case-control study

**DOI:** 10.1186/s12887-024-04909-6

**Published:** 2024-07-15

**Authors:** Omnia El-Kassas, Ayman Amer, Hesham Abdel-Hady, Tamer Abou-Elsaad

**Affiliations:** 1 Phoniatrics Unit, ORL Department, Faculty of Medicine, Mansoura University, Mansoura, 35516 Egypt; 2 Neonatology Unit, Pediatric Department, Faculty of Medicine, Mansoura University, Mansoura, Egypt

**Keywords:** Preterm, Readiness, Oral feeding skills, Scale, Full oral feeding, Algorithm for oral feeding

## Abstract

**Background:**

Oral feeding is a complex sensorimotor process influenced by many variables, making it challenging for healthcare providers to introduce and manage it. Feeding practice guided by tradition or a trial-and-error approach may be inconsistent and potentially delay the progression of oral feeding skills.

**Aim:**

To apply a new feeding approach that assesses early oral feeding independence skills of preterm infants in the neonatal intensive care unit (NICU). To prove its effectiveness, compare two approaches of oral feeding progression based on clinical outcomes in preterm infants, the traditional approach used in the NICU of Mansoura University Children Hospital (MUCH) versus the newly applied approach.

**Methods:**

A quasi-experimental, exploratory, and analytical design was employed using two groups, control and intervention groups, with 40 infants for the first group and 41 infants for the second one. The first group (the control) was done first and included observation of the standard practice in the NICU of MUCH for preterm oral feeding, in which oral feeding was dependent on post-menstrual age (PMA) and weight for four months. The second group (the intervention) included early progression to oral feeding depending on early assessment of Oral Feeding Skills (OFS) and early supportive intervention and/or feeding therapy if needed using the newly developed scoring system, the Mansoura Early Feeding Skills Assessment “MEFSA” for the other four months. Infants in both groups were studied from the day of admission till discharge.

**Results:**

In addition to age and weight criteria, other indicators for oral feeding readiness and oral motor skills were respected, such as oral feeding readiness cues, feeding practice, feeding maintenance, and feeding techniques. By following this approach, preterm infants achieved earlier start oral feeding (SOF) and full oral feeding (FOF) and were discharged with shorter periods of tube feeding. Infants gained weight without increasing their workload to the NICU team.

**Conclusion:**

The newly applied approach proved to be a successful bedside scoring system scale for assessing preterm infants’ early oral feeding independence skills in the NICU. It offers an early individualized experience of oral feeding without clinical complications.

## Background

Every year, there are more than 15 million preterm infants worldwide. In most countries, the incidence of prematurity is increasing, with substantial long-term costs to infants, families, and society [1]. About one out of eight preterm infants require admission to NICU for the 1st days, weeks, and even months of their life [2].

With the recent advances in both medical and technological fields, the rate of survival for extreme preterm and medically complicated infants is increasing [3]. With the improved survival rate of preterm infants, a novel perception arises about oral feeding difficulties in those fragile infants and the importance of overcoming these difficulties [4, 5]. Once the clinical and life-threatening problems that those infants face are overcome, struggling with oral feeding difficulties begins [6].

Ideally, for all hospitalized patients, after a good analysis of the clinical symptoms and making a differential diagnosis for the potential causes, clinicians draw a plan for assessing and managing the clinical issues. In NICUs, a multidisciplinary medical team works hand in hand to enforce the infants’ ability to achieve safe and independent oral feeding. This team comprises neonatologists, neonatal nurse practitioners, neonatal nurses, and feeding specialists, i.e., lactation consultants, neonatal nutritionists, occupational therapists (OT), and phoniatricians/speech therapists [7].

Preterm infants are exposed to multiple stressors in the prenatal and postnatal periods, leading to cumulative stress exposure, which primarily affects the achievement of successful oral feeding, and more than 70% of those infants experience difficulties with oral feeding progression [8]. Cumulative stress exposure leads to modifying glucocorticoid genes and altering cortisol reaction to external environmental stimuli, which may impair the neurological and behavioral development required for efficient oral feeding skills (OFS) [8]. Tube feeding is done to provide full newborn nourishment despite its known risks and negative impact on feeding abilities, as it promotes a protracted stay in the NICU and postpones oral feeding [9].

In an infant, swallowing development is modified and impacts the development of other organ morphology and other functions of the aero-digestive system, such as breathing and speech. Any obstacle in this evolution may delay the development of other oral-motor functions such as babbling, language, and speech production. Thus, early and appropriate assessment and intervention in infants are required to limit the adverse functional effects of dysphagia on the aero-digestive functions [9].

Clinical assessment is an integral part of evidence-based management of neonatal feeding problems. Clinical assessment helps the phoniatrician/speech pathologists to put a primary framework or profile of the possible cause of the feeding problems, know the parent’s understanding of the problem, identify oral feeding readiness, make a differential diagnosis for the causes, and determine the importance of multidisciplinary management [10].

If any deficits have been noted during the assessment, then a potential critical program for intervention dependent on assessment findings will be applied. In healthy and clinically stable preterm infants, “feeders and growers,” if we observe any deficit, it is primarily due to immaturity of true oro-pharyngeal dysphagia, especially if assessment takes place before term gestation [11].

Hence, feeding and swallowing difficulties are caused by multiple factors. Interventions for motor and sensory oral feeding problems followed a multifactorial framework. Several intervention strategies have been observed to shorten the transition time to achieve full oral feeding (FOF) in preterm infants [12]. The goal of the treating phoniatrician/speech pathologists is to plan for efficient and safe oral feeding, depending on the infant’s cues, using different compensatory measures, and to decide if extra evaluation is required [13].

There is a high prevalence of prematurity in Egypt, from 2.4% in 2011 to 4.7% in 2015, with the highest rate during 2013 (5.3%). Regarding fetal outcome, 61.3% of infants developed a poor fetal outcome, including; (fetal death and ICU admission), while 38.7% of infants had good fetal outcomes (alive & well), contributing to neonatal feeding difficulties [14].

No existing data on feeding difficulties in preterm infants are available in Egypt. Unfortunately, there are no definitive criteria to guide decisions about when and how to progress oral feeding in preterm infants, and feeding practices in the NICU are highly variable. There is no specific policy for initiating oral feeding, but it mostly depends on GA and weight criteria. In addition, phontricians have a limited role in feeding services in NICUs. Accordingly, there is an urge to study the transition to oral feeding in preterm infants. Therefore, we conducted this research to investigate the transition from nasogastric (NG) feeding to oral feeding in preterm infants in our locality.

### Aim

This study aims to compare two approaches of oral feeding progression based on clinical outcomes in preterm infants: the traditional approach used in the neonatal intensive care unit (NICU) of Mansoura University Children’s Hospital (MUCH) vs. the newly developed scoring system, the MEFSA [15].

## Subjects and methods

### Subjects

A quasi-experimental, exploratory, and analytical design was employed using two groups. All preterm infants who met the inclusion criteria were divided into two groups. The first group (the control) was done first and included observation of the standard practice in the NICU of MUCH for preterm oral feeding, in which oral feeding was dependent on PMA and weight for four months. The second group (the intervention) included early progression to oral feeding depending on early assessment of OFS and early supportive intervention and/or feeding therapy if needed using the newly developed scoring system, the Mansoura Early Feeding Skills Assessment “MEFSA” [15] for the other four months. Infants in both groups were studied from the day of admission till discharge.

The participants were the phoniatrician team of the Phoniatrics unit, the otorhinolaryngology (ORL) department of Mansoura University Hospitals (MUH), and attending neonatologists and practitioner nurses from the Neonatal Intensive Care Unit (NICU) of Mansoura University Children’s Hospital (MUCH).

Preterm infants < 37 weeks gestation who did not receive oral feeding and were diagnosed by the attending neonatologist as clinically stable to initiate oral feeding were included in the sample. Infants that presented at least one of the following conditions were excluded from the study: a known congenital or chromosomal disease, Cardiac malformation, infants who developed bronchopulmonary dysplasia, gastrointestinal problem (intestinal obstruction, feeding intolerance), head and neck malformation (cleft lip and palate), infants with intracranial hemorrhage or a surgical condition at the time of the study.

Several semi-structured, face-to-face meetings with NICU attending neonatologists and nurses responsible for feeding were done: (1) Firstly, open questions were asked about their opinion on the characteristics of preterm infants to start oral feeding, on interventions that promote the transition to oral feeding, and on the existence of a guideline for oral feeding. (2) Then, the principles of the study, the developed score, and follow-up sheets were discussed with them to follow the transition protocols and fulfill follow-up sheets. The sheets were blindly fulfilled to decrease the bias, i.e., the NICU nurses fulfilled the follow-up sheets, and every meal had a separate sheet to avoid the nurse copying the previous meal observation as each meal is different. (3) Finally, the needed outcomes (SOF, FOF, discharge characters, periods of tube feeding, weight gain, and workload to NICU team) are compared between the two groups.

### Methods

The study was carried out in three stages,

#### Firstly

Observing the standard practice in the NICU of MUCH for preterm oral feeding. The traditional oral feeding approach in the NICU follows a non-individualized system and considers feeding a task. Also, it ignores that each infant has capabilities that must be respected and supported. In this approach: (a) The NICU team depends mainly on PMA (36 weeks of GA) and weight criteria (1600 g), ignoring other criteria to initiate oral feeding. (b) This traditional approach supports a volume-based regimen. It follows a rigid scheduled system with the main focus of emptying the bottle. The success is measured by how much an infant ingests. (c) Caregivers adhere to wrong behavioral patterns to accomplish intake without regard to what the infant communicates. These wrong behaviors include (1) using the nipple as an arousal tool, (2) laying the infant back to increase the milk flow, (3) placing the nipple in the mouth without waiting for infant readiness, (4) feeding while the infant is tired, (5) poor positioning, (6) lack of postural support and (7) use of high flow rate nipple that is not suitable to those fragile infants. (d) The NICU team ignores the idea that sucking power is related to GA and that sucking ability improves with increasing GA. (e) Also, the NICU team misunderstands the difference between sucking patterns in full-term versus preterm infants. The sucking pattern in premature infants is higher in frequency, lower in amplitude, and weaker in power than in full-term infants [16]. So the NICU team claims that the pattern in preterm, which is typical for age, is having a week or no sucking and introducing NG feeding. (f) The NICU team misinterprets infant readiness behavioral cues, hunger cues, and subtle distress cues during feeding.

#### The second phase

Discusses the principles of the newly developed approach. The newly designed approach follows an assessment of the MEFSA score [15]. This approach supports the hypothesis of early assessment of OFS and early individualized support and feeding therapy if needed. It is a cue-based feeding approach. Caregivers must respect the idea that the feeder is a part of the feeding system and that both the feeder and the infant co-regulate feeding. This approach hopes infants become successful feeders, not just successfully fed. To practice functional oral feeding: (a) Initiate oral feeding once the infant is clinically stable and off the ventilator and/or CPAP. The functioning of the GIT system must first be proved by passing stool, audible intestinal sound, and enteral feeding tolerance. (b) Follow cue-based or infant-based technique that allows the infant to communicate. Caregivers watch, listen, observe, interpret, and respond to the infant’s language. Consequently, according to the infant’s communication cues, strategy is modified. (c) Use feeding competencies and assess the infant from moment to moment during feeding. Observe for (1) ability to organize oral motor function, (2) maintain engagement in feeding, (3) coordinate suck-swallow-breath, and (4) maintain stability. (d) Most of those fragile preterm infants required individualized therapy. They aim to support and help premature infants acquire the skills for efficient oral feeding. As such, it is a smoother transition and less stressful infant’ adaptation.

#### Lastly

The new approach using the MEFSA score should be applied and confirmed as effective by comparing the outcomes between the standard NICU approach and the newly developed approach using MEFSA [15].

### Summary of the MEFSA approach

The MEFSA is an 85-item observational measure of OFS. It follows a cue-based feeding regimen and includes three main sections: pre-feeding, during-feeding, and post-feeding. Moreover, recommendations are given to support preexisting feeding skills until systems are sufficiently mature for oral feeding. Interventions to facilitate the acquisition of OFS can also be recommended. Finally, the MEFSA ends with a plan section for further follow-up assessment. The MEFSA sections were scored with the highest score gained at every item, indicating the best infant’s oral feeding performance.

## Results

The study sample consisted of 81 preterm infants admitted to the NICU. 40 infants were assigned to the control group, and the following 41 infants were assigned to the intervention group. The study design includes two blind groups. The inclusion and exclusion criteria were applied for all preterm infants sharing in the study. To minimize the influence of weight and GA, the preterm infants were divided into subgroups according to prematurity class and birth weight categories. There was no statistical difference in these subgroups regarding GA and birth weight, as shown in (Table 1). Also, according to weight percentile age, the intervention group showed more small infants for gestational age (SGA) and large infants for gestational age (LGA).

Furthermore, clinical stability, the need for a ventilator and/or CPAP, and tolerance to enteral feeding are essential factors in initiating oral feeding in preterm infants. There was no significant difference regarding these previous factors.

**Table 1:** Demographic data of the studied groups (*N* = 81)

Parameters	Intervention group *N* = 41	Control group *N* = 40	Test of significance
Gestational age			
*(days)* *(weeks)*	229.8 ± 13.8 32.8 ± 1.97	220.6 ± 18.4 31.5 ± 2.63	t = 2.56 *P* = 0.01
Prematurity class			MC = 6.1 *P* = 0.04
Mild preterm (36- Very preterm (32 w- Extremely preterm (≤ 28 w)	27 (65.9%) 11 (26.8%) 3 (7.3%)	15 (37.5%) 18 (45%) 7 (17.5%)	
Birth/admission weight *(gm)*	1747.0 ± 519.1	1430.1 ± 433.6	t = 2.98 *P* = 0.004
Body weight categories			MC = 4.58 *P* = 0.08
Normal LBW VLBW ELBW	4 (9.8%) 21 (51.2%) 13 (31.7%) 3 (7.3%)	2 (5%) 12 (30%) 24 (60%) 2 (5%)	
Weight percentile age			X^2^ = 1.71 *P* = 0.19
Appropriate for age Not appropriate for age Small for age Large for age	34 (82.9%) 7 (17.1%) 4 (9.8%) 3 (7.3%)	37 (92.5%) 3 (7.5%) 3 (7.5%) 0	
Needing ventilator and/or CPAP			X^2^ = 2.19 *P* = 0.13
No Yes	13 (31.7%) 28 (68.3%)	7 (17.5%) 33 (82.5%)	
Off ventilator and/or CPAP and showing tolerance to enteral feeding PMA			t = 1.2 *P* = 0.25
*(days)* *(weeks)*	233.9 ± 15.3 33.1 ± 2.18	228.9 ± 17.6 32.7 ± 2.51	

The results of this study, shown in (Table 2), supported the hypotheses of the newly developed approach. Infants in the intervention group started oral feeding (SOF) earlier, 21.5 days after admission, and fewer than 15 days after being clinically stable than the other group, with a mean of 10 days younger in the intervention group. The mean SOF weight was 1683.7 ± 429.9 gm for the intervention group and 1750.1 ± 354.5 for the control group.

In the intervention group, infants progressed to oral feeding more smoothly and rapidly, with a median transition period of 2 days (2–9) and 3 (2–12) for the other group. In the intervention group, infants reached FOF 12 ± 3.2 days younger. The mean FOF weight was 1750.5 ± 437.5 gm for the intervention group and 1856 ± 377.7 gm for the control group.

As a result of early experience with oral feeding, 27 out of 41 infants in the intervention group needed NG feeding, while all the control group infants needed NG feeding. Furthermore, the intervention group had a median of 20 days of parental nutrition.

Infants in the intervention group were discharged to home 16 days earlier than the control group. Infants released 14.8 ± 4.8 days younger in the intervention group. The mean discharge weight was 2049.9 ± 457.1 gm for the intervention group and 1919.4 ± 375.7 gm for the control group.

**Table 2:** Oral feeding, Ryle feeding, and discharge characters for the studied groups (*N* = 81)

Parameters	Intervention group *N* = 41	Control group *N* = 40	Test of significance
**Oral feeding characters in the studied group**			
The period from birth/admission to SOF *(days)*	3 (0–24)	24.5 (2–66)	Z = 6.41 ***P*** ≤ 0.001*
The period between being stable, off Ventilator and / or CPAP to SOF *(days)*	1 (0–10)	16 (2–37)	Z = 6.39 ***P*** ≤ 0.001*
SOF weight *(gm)*	1683.7 ± 429.9	1750.1 ± 354.5	t = 0.76 *P* = 0.45
SOF PMA *(days)* *(weeks)*	236.2 ± 13.1 33.74 ± 1.87	246.3 ± 15.9 35.18 ± 2.27	t = 3.11 ***P*** = 0.003*
Period of transition from SOF to FOF *(days)*	2 (2–9)	3 (2–12)	Z = 3.54 ***P*** ≤ 0.001*
FOF weight *(gm)*	1750.5 ± 437.5	1856 ± 377.7	t = 1.2 *P* = 0.25
FOF PMA *(days)* *(weeks)*	239.1 ± 12.5 34.15 ± 1.79	251 ± 15.7 35.85 ± 2.24	t = 3.8 ***P*** ≤ 0.001*
**Ryle feeding characters in the studied groups**			
Using/needing Ryle			FET ***P*** ≤ 0.001*
No Yes	14 (34.1%) 27 (65.9%)	0 (0%) 40 (100%)	
Transition from Ryle to SOF *(days)*	4 (1–24)	22.5 (2–66)	Z = 5.47 ***P*** ≤ 0.001*
Total parenteral nutrition duration *(days)*	5 (2–26)	25 (4–76)	Z = 5.73 ***P*** ≤ 0.001*
**Discharge characters in the studied group**			
Discharge weight *(gm)*	2049.9 ± 457.1	1919.4 ± 375.7	t = 1.4 *P* = 0.17
Discharge PMA *(days)* *(weeks)*	249.6 ± 12.7 35.65 ± 1.81	254.4 ± 17.5 36.34 ± 2.5	t = 1.4 *P* = 1.7
Total period of stay in NICU *(days)*	17 (5–56)	33 (7–80)	Z = 3.55 ***P*** ≤ 0.001*

Data expressed as mean ± SD, median (minimum–maximum), or number (%)

t: independent samples -t-test

Z: Mann Whitney test

FET: Fisher’s Exact Test

*: significant *p* ≤ 0.05

Offering preterm infants early oral feeding experience may raise concerns that tremendous energy will be expended, resulting in slower weight gain. Table 3 shows that both groups had satisfactory weight gain in this study. Additionally, there was no significant difference in weight gain across groups except in the period from birth/admission to SOF. The study of Haseli et al. [17] reported the known hypothesis: neonatal weight loss (NWL) in the first few days of life is a common phenomenon in which infants lose weight after birth before gaining weight. The rate of NWL has been reported as 4–7%. In the intervention group, the period from birth/admission to SOF was a median of 3 days (the 1st few days of NWL), while it was 24.5 days in the control group.

**Table 3:** Change in weight in the period from birth/admission till discharge from NICU in the studied group (*N* = 81)

Parameters	Intervention group *N* = 41	Control group *N* = 40	Test of significance
Change in weight per day in the period from birth/admission to SOF	0 (-150–50)	12 (-30–81)	Z = 4.42 *P* ≤ 0.001*
Change in weight per day in a period of transition from SOF to FOF	18 (-28–168)	25 (8–140)	Z = 0.82 *P* = 0.41
Change in weight per day in the period from FOF till discharge	25 (-9–103)	20 (0–100)	Z = 1.66 *P* = 0.09

Data expressed as median (minimum-maximum)

Z: Mann Whitney test

*: significant *p* ≤ 0.05

This study showed in (Table 4) a significant association between BW, SOF-weight, and FOF-weight in the control group. This means infants were not offered oral feeding till reaching a certain weight. On the contrary, there was no significant association in the intervention group. Furthermore, there was a significant association between GA, SOF-PMA, and FOF-PMA in both groups but with lower significance in the intervention group. In the control group, offering oral feeding depends mainly on PMA. In addition to age and weight criteria, other indicators for oral feeding readiness and oral motor skills must be respected, such as oral feeding readiness cues, feeding practice, feeding maintenance, and feeding techniques.

**Table 4:** Association between BW, SOF weight, FOF weight, and between GA, SOF PMA, FOF, and PMA in the studied group

Weight (gm)				
**Parameters**	**BW**	**SOF weight**	**FOF weight**	**Test of significance**
Intervention group (*n* = 41)	1747.0 ± 519.1	1683.7 ± 429.9	1750.5 ± 437.5	F = 0.27 *P* = 0.76
Control group (*n* = 40)	1430.1 ± 433.6	1750.1 ± 354.5	1856 ± 377.7	F = 12.7 *P* ≤ 0.001*
**PMA** ***(weeks)***				
**Parameters**	**GA**	**SOF PMA**	**FOF PMA**	**Test of significance**
Intervention group (*n* = 41)	32.8 ± 1.97	33.74 ± 1.87	34.15 ± 1.79	F = 5.3 *P* = 0.006*
Control group (*n* = 40)	31.5 ± 2.63	35.18 ± 2.27	35.85 ± 2.24	F = 38.3 *P* ≤ 0.001*

Data expressed as mean ± SD

F: one-way ANOVA test

*: significant *p* ≤ 0.05

## Discussion/conclusion

Before SOF, assessing specific parameters without which the transition process would be compromised is essential. The physiological stability, including respiratory independence, heart rate, and oxygen saturation within the normal range, was considered a prerequisite for suck-swallow-breathing (SSB) coordination in preterm infants. The presence of sucking reflexes and swallowing and, more critically, SSB coordination is an indicator of oral feeding readiness. The overall appearance, such as skin color, temperature, and muscle tone, are indicators that cannot be neglected as they translate the infant’s clinical condition and hemodynamic stability, which are essential for preterm infants to participate during feeding without excessive energy expenditure actively. The infant should show readiness; hunger signals must be awake and have a suitable suction power. The hypotonic or sleeping infant without signs of readiness should be fed by tube.

The stimulation of oral motor skills was also considered necessary in the achievement of oral feeding in preterm infants, such as sucking training, swallowing exercise, endurance exercise NNS with a pacifier, OS, and kangaroo care. Also, repositioning the infant and adjusting the flow rate was essential.

Infant cues before, during, and after meals are decisive factors in the infant’s ability to maintain organization, self-regulation, and good performance. Infant tolerance to nursing routine care, such as changing the diaper, bathing, or suction of secretions, should be registered and carefully planned before the meal to preserve energy for the complex task of oral feeding.

Even if subtle, the clinical stress signals expressed by preterm infants during the meal are indicators to stop feeding, give the baby rest, and apply the necessary support to help self-regulation. Pacing and regulation help the baby to breathe, maintain physiological stability, and prevent fatigue. Also, decreasing external stimuli, such as light and noise, would help reduce stress and provide a suitable feeding environment.

The results of this study proved the essentiality of having guidelines for oral feeding in preterm infants based on scientific evidence and the early support of oral feeding skills. The outcomes of the newly applied approach using the MEFSA score proved to be a successful oral feeding progression approach in preterm infants in the NICU.

This study revealed that early assessment and supportive interventions decreased the number of days to reach FOF in preterm infants, leading to earlier hospital discharge. The latter result agrees with Younesian et al. [18], who found that FOF was achieved significantly earlier in the infants in the experimental group who received early oral stimulation than in the controls who were not offered any stimulations. Likewise, the length of hospitalization was significantly shorter in the experimental group than in the control group. The two groups showed no significant difference in terms of weight gain. Moreover, Liu et al. [19] found a shorter transition period from SOF to FOF, earlier SOF, and earlier discharge with shorter NICU stay in the experimental group who received a stimulation program than in the control group who did not offer stimulation. Also, earlier commencement/introduction of oral feeding was reported by Kamhawy et al. [20] study, which showed an accelerated transition to nipple feeding and earlier discharge in the intervention group who received non-nutritive sucking (NNS). Furthermore, Lyu et al. [21] reported that PMA and weight at FOF were significantly lower in the experimental group who received oral stimulation (OS) added to routine care with a shorter time from initiation of SOF to FOF compared to controls who received only standard care. No significant differences existed in the length of hospital stay or weight gain rate. Additionally, compared to control infants who received routine NICU care, infants in the experimental group receiving the intervention had shorter and easier transition periods to FOF [22, 23].

**Fig. 1:**
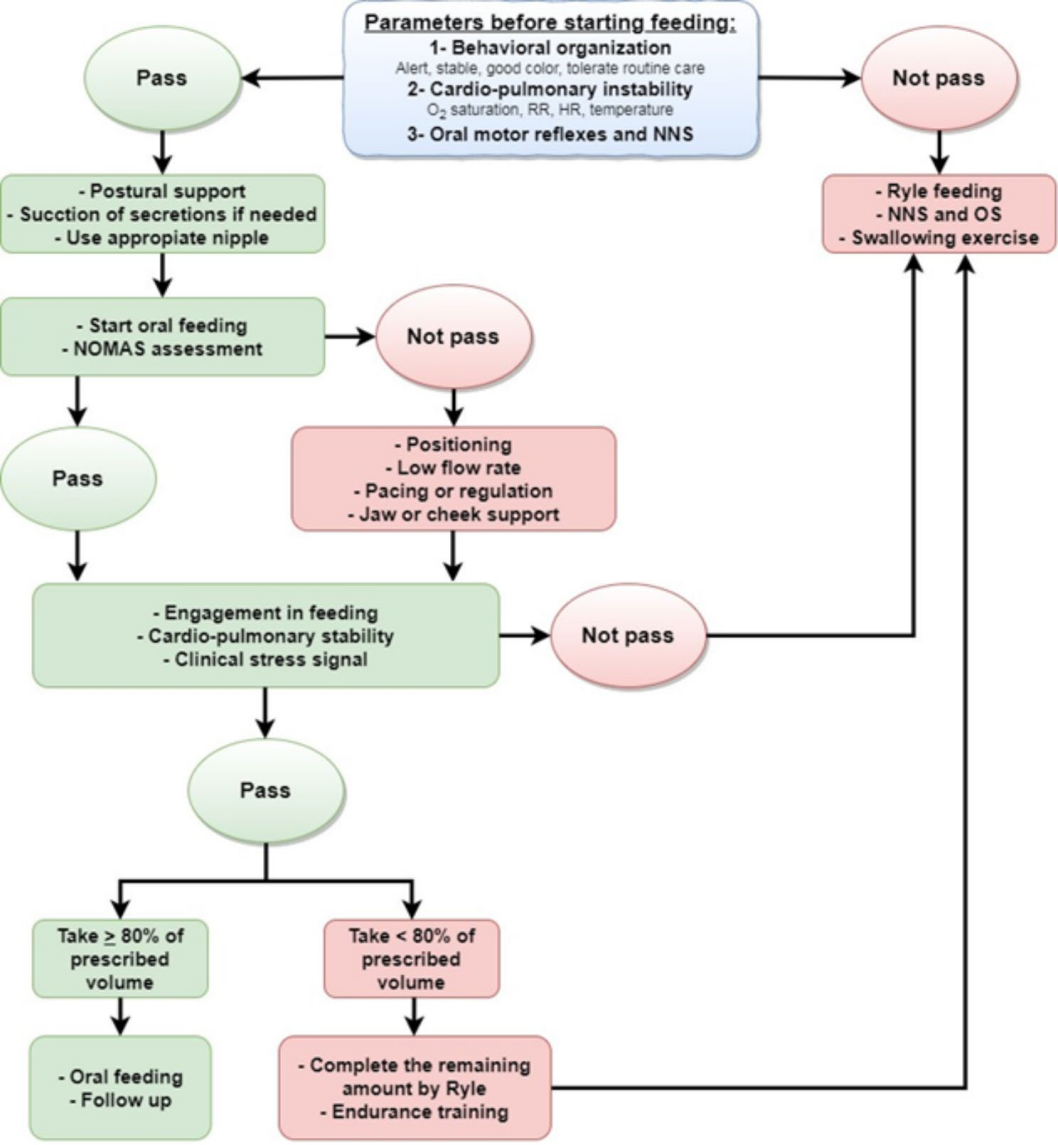
An algorithm for assessing and supporting oral feeding in preterm infants following MEFSA score

Finally, Fig. 1 represents an algorithm for oral feeding. This algorithm is the best contribution of this study to assessing and supporting oral feeding in preterm infants.

## Data Availability

The datasets used and/or analyzed during the current study are available from the corresponding author upon reasonable request.

## Abbreviations

BW: Birth Weight
CPAP: Continuous Pulmonary Air Pressure
FOF: Full Oral Feeding
GA: Gestational Age
GIT: Gastrointestinal Tract
ICU: Intensive Care Unit
MEFSA: Mansoura Early Feeding Skills Assessment
MUCH: Mansoura University Children’s Hospital
NG: Nasogastric Feeding
NICU: Neonatal Intensive Care Unit
NNS: Non-Nutritive Sucking
NWL: Neonatal Weight Loss
OFS: Oral Feeding Skills
ORL: Otorhinolaryngology
OS: Oral Stimulation
OT: Occupational Therapists
PMA: Premenstrual Age
SOF: Start Oral Feeding
SGA: Small for Gestational Age
LGA: Large for Gesational Age
